# BRAF V600E-mutated metastatic Wilms tumor with complete response to targeted RAF/MEK inhibition: a post-treatment follow-up case report

**DOI:** 10.3389/fonc.2026.1819524

**Published:** 2026-05-07

**Authors:** Iliani Slika, Carlene K. Edwards, Kenneth J. Cohen, Alan D. Friedman, Christine A. Pratilas, Patience Odeniyide

**Affiliations:** 1 Department of Oncology, Sidney Kimmel Comprehensive Cancer Center (SKCCC), Johns Hopkins University School of Medicine, Baltimore, MD, United States; 2 Department of Pediatrics, Johns Hopkins University School of Medicine, Baltimore, MD, United States

**Keywords:** BRAF V600E mutation, BRAF/MEK inhibitor, case report, targeted therapy, Wilms tumor

## Abstract

Wilms tumor (WT) is the most common pediatric renal malignancy. The standard treatment approach of surgical resection and cytotoxic chemotherapy has led to excellent outcomes for most patients, particularly those with favorable histology, with a 4-year overall survival of more than 90%. However, cases of multiply relapsed WT have dismal outcomes with limited effective options. Little is known about the role of precision-targeted therapy in recurrent Wilms tumor. We previously reported a pediatric patient with recurrent pulmonary metastatic favorable histology WT with a BRAF V600E mutation, who was successfully treated with BRAF/MEK inhibition. Here we provide an update on this patient’s course, seven years from the identification of the second pulmonary metastatic relapse and initiation of genomically informed treatment. Following five years of treatment, the patient now remains disease-free for two years following discontinuation of targeted therapy, which was well tolerated without major adverse events. This report presents the first case of long-term survival after targeted therapy for multiply relapsed BRAF V600E WT.

## 1 Introduction

Nephroblastoma or Wilms tumor (WT) represents more than 90% of all primary renal malignancies in children 0–7 years of age (1). It has an excellent prognosis for low-stage favorable histology (FH), with a 90-100% 4-year overall survival (OS) (2–5), compared to those with advanced stage (OS rates reaching 88.9%) (6) and/or anaplastic disease (OS rates ranging from 85.7% for focal anaplastic disease (7) to 73.7% for stage II-IV diffuse anaplastic WT (DAWT) (8). Although for patients with stage IV favorable histology WT and pulmonary only metastatic disease, 4-year OS rates remain high at 95.6% (9), OS rates for relapsed FHWT are around 80% (10, 11). There are no established treatment guidelines for patients with Wilms tumor in second or greater relapse.

Herein, we present the seven-year follow-up of a previously described pediatric case (12) involving multiply relapsed epithelial-predominant Wilms tumor (WT) with overlapping metanephric adenoma features harboring the BRAF V600E mutation. After relapse with second-line chemotherapy, the patient was treated with combined BRAF and MEK inhibition (dabrafenib and trametinib), achieving a sustained complete remission. The patient remains disease-free seven years after the second recurrence and two years following cessation of targeted therapy. This case highlights the important role of molecular profiling and BRAF-targeted therapy in relapsed WT and represents, to our knowledge, the first report of durable long-term remission with BRAF V600E-directed treatment in this setting.

## 2 Case description

**Figure 1:**
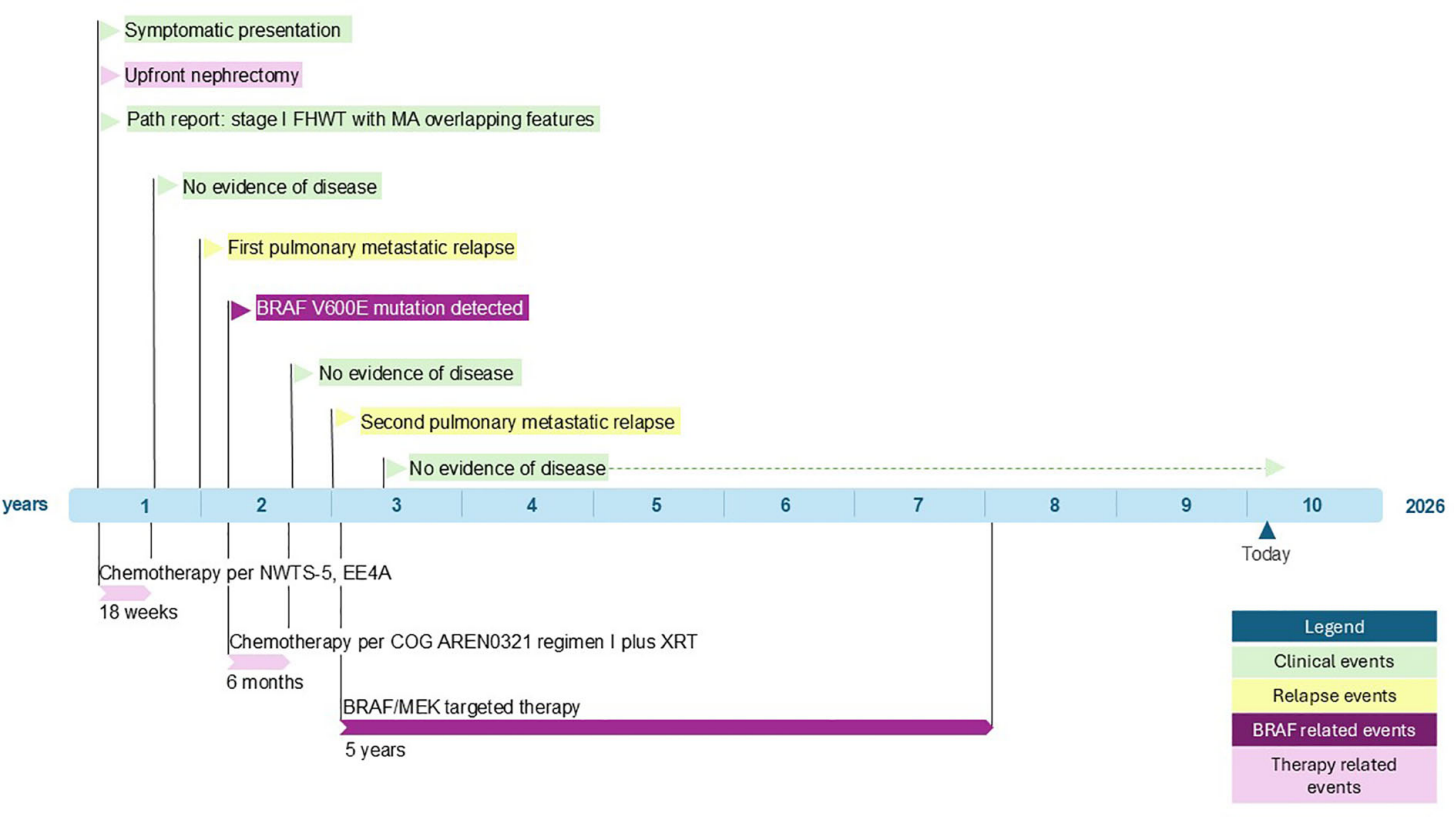
Timeline of clinical course, therapeutic interventions, and outcomes in a pediatric patient with BRAFV600E-mutated Wilms tumor.

A 6-year-old male patient presented with hematuria, nausea, and right flank pain. Diagnostic ultrasound and abdominal computed tomography (CT) scan confirmed the presence of a right renal mass, and the patient underwent right radical nephrectomy. Histopathologic analysis classified the tumor as stage 1 WT with favorable histology, characterized by triphasic elements and epithelial predominance, with overlapping features of metanephric adenoma. The patient received an 18-week course of therapy with vincristine and dactinomycin according to the National Wilms Tumor Study 5 (protocol 4941), Regimen EE-4A, and had no evidence of disease at the completion of chemotherapy (**Figure 1**).

Five months post-completion of treatment, a chest CT scan revealed unilateral solitary pulmonary metastatic disease, without local tumor recurrence. An excisional biopsy confirmed metastatic WT, and next-generation sequencing (NGS) revealed a BRAF V600E mutation without additional actionable alterations. The patient received salvage chemotherapy with vincristine, doxorubicin, cyclophosphamide, and etoposide per Children’s Oncology Group protocol AREN0321 Regimen I and radiation therapy, again with no evidence of disease at the end of planned therapy. Surveillance CT imaging three months later uncovered a second metastatic pulmonary relapse, consisting of at least ten small but measurable nodules. NGS and immunohistochemistry analysis of both the primary renal mass and the first pulmonary metastasis confirmed the presence of the BRAF V600E mutation, suggesting a clonal origin traced to the original tumor rather than the possibility of acquiring the BRAF mutation at the time of first recurrence. This increased the confidence that the new pulmonary nodules, too, would express BRAF V600E. Therapeutic considerations included high-dose chemotherapy with autologous stem cell transplant, but after consideration of several factors, including the expected toxicities and the opportunity for a novel precision-driven approach, a decision was made to initiate combined BRAF (dabrafenib) and MEK inhibitor (trametinib) therapy. The treatment regimen consisted of trametinib 0.5 mg daily for 3 days per week and 0.75 mg daily for 4 days per week, in combination with dabrafenib 75 mg in the morning and 50 mg in the evening. This regimen was based on pediatric dosing established oin the NCT02124772 clinical trial (13) (dabrafenib 5.25 mg/kg/day, age <12 years and trametinib 0.025 mg/kg/day, age >/= 6 year) administered in 28-day cycles. The patient tolerated treatment well, had no missed doses, and experienced only minor adverse effects that required brief treatment interruptions. Specifically, the patient experienced six episodes of treatment-related pyrexia, each of which resolved after a 48-hour interruption of therapy, after which treatment was successfully resumed without further issue. Eight weeks after the initiation of therapy, there was a partial resolution of pulmonary nodules on imaging which evolved to a complete radiographic response after 16 weeks of treatment (12).

Relapse surveillance included interval chest, abdomen, and pelvis CT scans every four months. Plasma-based BRAF V600E cell-free DNA (cfDNA) testing was used as a noninvasive adjunct to radiographic monitoring, beginning 7 months after initiation of BRAF/MEK inhibitor therapy, with no pre-treatment baseline sample available. cfDNA testing was repeated every 4–6 months both during treatment and after its discontinuation. All tests were negative (did not detect BRAF V600E in circulation) and correlated with CT imaging demonstrating no evidence of disease. Dabrafenib and trametinib therapy was continued for nearly five years prior to cessation. Presently, the patient is two years post-discontinuation of treatment and remains disease-free (**Figure 1**).

## 3 Discussion

### 3.1 Genetic landscape of WT

**Figure 2:**
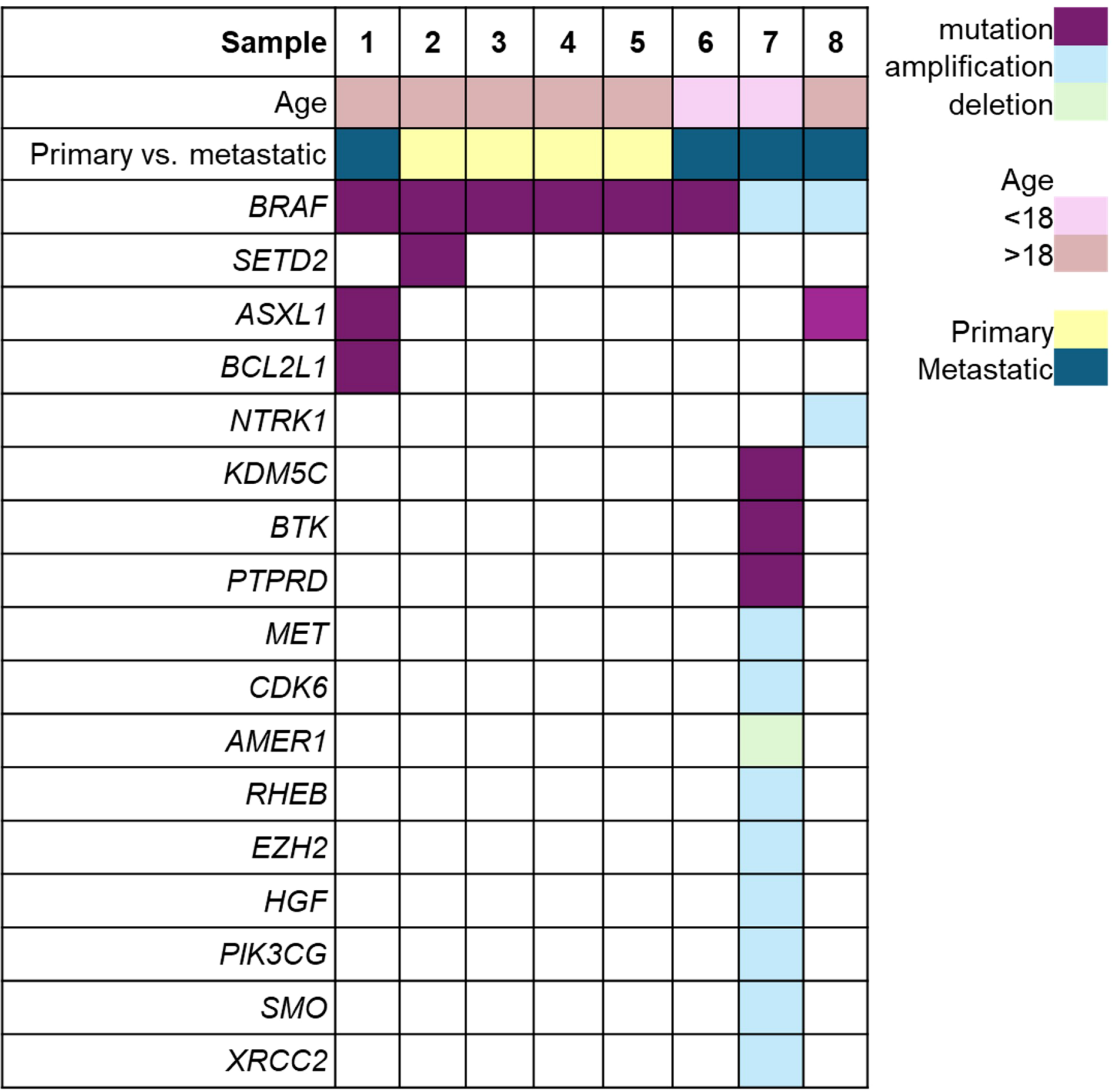
Oncoprint presenting the mutational profile of the 8 out of 307 WT samples in which BRAF gene alterations were detected in the GENIE Cohort 19. Sample 6 represents the patient from our case report.

The most commonly observed genomic alterations in WT include mutations in *TP53, CTNNB1, DROSHA, MYCN*, and *WT1*, and copy number alterations in *AMER1, KMT2B, HIST2H3D, AKT2*, and *MYCN* (14). BRAF V600E mutations, initially described in 2002 (15) and seen in a high proportion of melanoma and several other cancers (including colorectal, thyroid, and primary CNS malignancies), are observed at relatively low frequencies in nearly all cancer types, and have been detected in 2% of WT cases in the GENIE cohort which profiled 307 WT samples (14). Notably, BRAF alterations (mutations and amplifications) were observed at similar frequencies in metastatic and primary WT samples in this dataset (**Figure 2**).

Metanephric adenoma (MA), a renal tumor with embryonal histological characteristics, harbors BRAF V600E mutations in 85% or more of cases (16, 17). BRAF V600E mutations have also been detected in pediatric MA tumors (18) as well as metanephric stromal tumors (MTS) (19, 20). Although BRAF V600E is rarely detected in WT, a subset of epithelial-predominant WT cases with overlapping MA features has been reported to carry BRAF V600E mutations. Wobker et al. were the first to report four cases of both pediatric and adult WT with MA resembling areas harboring BRAF V600E mutations (21). This observation is further supported by a study of 14 adult WT patients, which identified BRAF V600E mutations in 36% of cases (22). All of these cases displayed histological areas resembling MA. Pan et al. have also reported a case of overlapping WT-MA with a BRAF V600E mutation that clustered with typical MAs rather than epithelial WT in differential gene expression analysis (23). Analogous findings were reported in a study of nine epithelial-predominant WT samples, in which Xu et al. showed that most tumors (8/9) clustered closely with MAs in genetic analyses and displayed similar expression patterns (24). These findings suggest that a molecularly defined subset of epithelial-predominant WT with metanephric adenoma–like features may harbor BRAF V600E mutations and raises the possibility that these tumors may represent a biologically distinct subset, with potential implications for diagnostic classification and selection of targeted therapy.

### 3.2 Advances in BRAF targeted therapy

BRAF inhibitor therapy with vemurafenib (the first of the class I BRAF or BRAF monomer inhibitors) demonstrated clinical efficacy in unresectable or metastatic melanoma harboring BRAF V600E mutation, leading to FDA approval for this indication in 2011 (25, 26). This clinical advance transformed the landscape of genome-driven precision oncology for BRAF V600E-mutated solid tumors (27). To date, several class I BRAF inhibitors, including dabrafenib and encorafenib, have also demonstrated clinical potency as BRAF inhibitors (28, 29). Dabrafenib monotherapy was approved by the FDA in 2013 for the treatment of unresectable or metastatic melanoma with BRAF V600E mutation (30) (**Table 1**).

**Table 1:** FDA-approved BRAF-targeted therapies and their corresponding indications.

Tumor type	Frequency of *BRAF* mutations	FDA approved *BRAF* inhibitor-based therapies	Year of FDA approval
Melanoma	36%	Vemurafenib	2011 (26)
		Dabrafenib	2013 (30)
		Dabrafenib plus trametinib	2014 (31)
		Vemurafenib plus cobimetinib	2015 (32)
		Encorafenib plus binimetinib	2018 (33)
Non-small cell lung cancer	5.6%	Dabrafenib plus trametinib	2017 (34)
		Encorafenib plus binimetinib	2023 (35)
Anaplastic thyroid carcinoma	40%	Dabrafenib plus trametinib	2018 (36)
Erdheim Chester disease	11.4%	Vemurafenib	2017 (37)
Colorectal cancer	12%	Encorafenib plus cetuximab	2020 (38)
Low grade glioma	14.6%	Dabrafenib plus trametinib*	2023 (39)
		Tovorafenib**	2024 (40)
Pediatric and adult non resectable and metastatic solid tumors	N/A	Dabrafenib plus trametinib	2022 (41)

Tumor type refers to the general category of the tumor for which the drug has FDA approval, although more specific indications may exist within each category. Frequencies of *BRAF* mutations for each tumor type were extracted from the AACR Project GENIE 19.0-public database (January 2026), which includes approximately 271,000 sequenced tumor samples from over 227,000 patients (14).

*FDA approval is limited to pediatric patients only.

**Tovorafenib is a pan-RAF inhibitor targeting multiple RAF kinases, not a selective BRAF inhibitor.

Class I RAF inhibitor-induced paradoxical activation of the RAS-RAF-ERK pathway (in non-V600E expressing normal tissues) was identified early as a barrier to their use as monotherapy (42–45). In malignant tissues, ERK reactivation after BRAF inhibition is one mechanism by which tumors develop resistance to this class of drugs (46). In non-malignant tissues, BRAF monotherapy results in characteristic on-target, off-tumor toxicities, such as hyperproliferative skin disorders and secondary cancers (47). Concurrent MEK inhibition suppresses paradoxical ERK reactivation, rendering the combination of BRAF/MEK inhibition both more effective and associated with reduced treatment-related toxicity than monotherapy in some cases (48–50). The first successful combination of BRAF and MEK inhibitors was dabrafenib and trametinib, which received FDA approval in 2014 for unresectable or metastatic BRAF V600E/K-mutant melanoma (31). Besides melanoma, the FDA has approved combined dabrafenib and trametinib therapy for multiple BRAF-mutated malignancies, including non-small cell lung cancer (NSCLC) (34), anaplastic thyroid carcinoma (36), and pediatric low-grade glioma (39), primarily in the setting of BRAF V600E–mutated metastatic or advanced disease (**Table 1**). In 2025, the FDA approved tovorafenib, a type II RAF kinase inhibitor, for pediatric relapsed or refractory low-grade glioma harboring BRAF fusion or BRAF V600 mutation, providing an alternative treatment for BRAF fusion tumors that bypasses the RAF paradox caused by type 1 RAF inhibitors (40).

In 2022, eight years after the first FDA approval of combined BRAF/MEK inhibition for specific cancer types, the FDA granted the first histology-agnostic approval of BRAF and MEK inhibitor combination therapy for patients with unresectable or metastatic BRAF V600E-mutant solid tumors, excluding colorectal cancer (CRC), who have experienced disease progression following prior treatment and have no satisfactory alternative therapeutic options (41). Evidence for approval of dabrafenib plus trametinib histology agnostic use in adult and pediatric solid tumors with BRAF V600E mutations was based on the NCI-MATCH and ROAR trials in adults and the CTMT212X2101 (X2101) study in pediatric patients. It was also supported by findings from melanoma and NSCLC trials COMBI-d, COMBI-v, and BRF113928 (51). The NCI-MATCH trial, subprotocol H, specifically evaluated dabrafenib plus trametinib in adult patients with non-melanoma BRAF V600E-mutated solid tumors (52), and ROAR was a phase II study focused on rare cancers with BRAF V600E mutation (53). Overall, 167 patients (131 adults and 36 children) across 24 cancer types were included. The objective response rate (ORR) across all cancer types was 41% in adults and 25% in children. Responses were durable, with 78% lasting at least 6 months and 44% lasting 24 months or longer in the pediatric cohort, whereas in the adult trials, the median duration of response was as high as 31.2 months in the ROAR trial (53) and 25.1 months in the NCI MATCH study (52). Common treatment-related adverse events included pyrexia, nausea, constipation, vomiting, fatigue, rash, and headache (52, 53).

### 3.3 BRAF/MEK targeted therapy: current challenges and future directions

This paradigm shifting novel FDA approval mirrors the new era of precision oncology, in which treatment decisions are guided by molecular alterations rather than tissue of origin. Basket trials and tumor agnostic approvals have shown that targeting specific oncogenic drivers can provide effective therapies across diverse tumor types, accelerating drug development and expanding options for patients with rare or refractory cancers. As of 2025, there are eight additional FDA approved therapies for histology agnostic indications, including two immune checkpoint inhibitors (54, 55) and five targeted agents such as NTRK (56, 57), RET (58), and HER2 directed therapies (59).

To our knowledge, this case, reported three years before the U.S. histology agnostic approval, was the first reported case of pediatric patient with pulmonary metastatic epithelial-predominant Wilms tumor with a BRAF V600E mutation, achieving a complete response to RAF/MEK inhibition. Additionally, two cases of adults with metastatic epithelial-predominant WT with a BRAF V600E mutation have since been reported. One patient had a durable response (>24 months) to BRAF inhibition (60). The second patient received combined BRAF and MEK inhibitor and achieved a partial response but later was found to have a new KRAS G13D mutation as a mechanism of treatment emergent resistance (61). These three reported cases, including our patient, had metastatic disease that was macroscopically measurable and, in the adult patients, extensive with multiple metastatic sites. In our patient, the CT at the time of starting BRAF/MEK inhibitor therapy demonstrated at least 10, albeit relatively small, pulmonary metastases (12), indicating a lower tumor burden compared to the adult cases. Several other studies have also reported the efficacy of dabrafenib and trametinib dual therapy in patients with baseline measurable tumors that could be radiographically assessed, including the pediatric low grade glioma clinical trial NCT02124772 (13). In addition, adjuvant use of dabrafenib and trametinib in resected stage III melanoma was found to increase relapse free survival (62), suggesting potential activity in the setting of minimal residual disease following surgery. Due to the small number of reported cases of BRAF/MEK inhibitor therapy in WT patients, it is difficult to conclude whether the optimal response of our patient to this specific therapy is attributed to his lower tumor burden compared to the adult cases, or there are other factors that affect efficacy.

In the NCI MATCH trial, Subprotocol H, which served as the basis for FDA approval of dabrafenib plus trametinib for tissue-agnostic indications, a high degree of tumor mutational heterogeneity was reported and trends toward differences in patient outcomes were observed, although these did not reach statistical significance (52). The possibility of mutational heterogeneity playing a role in the efficacy of BRAF/MEK targeted therapy is also supported by the fact that BRAF/MEK inhibition is more effective in certain tumor types, such as melanoma, compared with others, such as colorectal cancer (63) - these differences may be attributable to activation of parallel pathways (i.e. PI3K), or upstream receptor activation (i.e. EGFR). Based on this information, we have reasonable grounds to support that BRAF/MEK inhibitor therapy can be highly effective in a heterogeneous population of macroscopically measurable tumors. However, more specific assumptions regarding the efficacy of BRAF/MEK inhibitor therapy in Wilms tumors with high mutational burden and particular microenvironmental factors cannot be confidently drawn due to the lack of supporting literature.

The difference in response to therapy between our patient and the two other reported WT patients treated with BRAF targeted therapy, raises additional questions regarding the optimization of therapeutic strategies. There is evidence that tumors harboring BRAF V600E mutations can either exhibit early resistance to BRAF/MEK dual inhibitor therapy or acquire secondary resistance after an initial response (64–66). Resistance may arise from alterations that are either dependent on or independent of the MAPK pathway. In addition to MAPK-driven mechanisms, resistance can also involve other RAF paralogs (CRAF, ARAF) and other complex pathways, including MET and the PI3K/AKT/mTOR signaling axis (41, 67). These findings highlight the need for further studies to optimize dosing regimens in both adult and pediatric patients, as well as to develop strategies to prevent or overcome secondary resistance.

A frequent challenge that arises following successful treatments with similar targeted therapies is the unknown consequences of discontinuation, and whether patients will experience rapid recurrence or other adverse outcomes. The treating team’s decision to continue therapy for 5 years was based on the therapeutic approach established in the NCT02124772 clinical trial for pediatric low-grade gliomas treated with dabrafenib and trametinib (13). Based on the paradigm of this study, and taking into consideration our patient’s multiply relapsed disease, we continued treatment until loss of benefit or unacceptable toxicity emerged. After a sustained period of disease control over several years was achieved, we weighed the continued risk/benefit ratio of treatment continuation versus discontinuation, and recognizing the absence of evidence based data to guide our decision, in shared decision making with the family, elected for discontinuation and close surveillance with imaging. Our experience parallels a recent report of the use of larotrectinib in pediatric patients with TRK fusion sarcomas, that assessed clinical outcomes of elective discontinuation of treatment on disease progression-free cases in a wait-and-see protocol (68).Of the 47 patients who had a complete initial response to larotrectinib and discontinued therapy after a median treatment duration of 14.7 months, 36% had disease progression and resumed larotrectinib, with 94% achieving disease control on reinitiation. Following the example of elective larotrectinib discontinuation within a wait-and-see protocol, we propose further histology-agnostic studies specifically designed to determine the optimal timing for targeted therapy treatment discontinuation after a complete response.

## 4 Conclusion

Here, we present a seven-year follow-up of the first reported pediatric patient with metastatic recurrent Wilms tumor who achieved a complete response to BRAF/MEK inhibition, treated for five years with elective discontinuation, with no evidence of disease two years after stopping. This case highlights the benefit of BRAF/MEK–targeted therapy across a broader spectrum of BRAF V600E-mutated tumors and provides evidence that sustained disease control may be achieved under 5 years of treatment and maintained for at least 2 years after treatment discontinuation. This therapeutic strategy may significantly alter the treatment landscape for several rare, aggressive, or recurrent malignancies in which conventional chemotherapy regimens are either insufficient or associated with substantial toxicity.

## Data Availability

Publicly available datasets were analyzed in this study. The data supporting the findings of this study are derived from a single clinical case and previously published with details regarding data accessibility (PMID: 32238401). In addition, publicly available comparative genomic data from the AACR Project GENIE database (cohort 19) were used in this study.
